# Traumatic chest wall pyomyositis presenting with mediastinitis

**DOI:** 10.1093/jscr/rjac503

**Published:** 2022-11-14

**Authors:** Ramanen Sugunesegran, Michael L Williams, Raymond Shi, Brendan Arnold, Philip J Davis

**Affiliations:** Department of Cardiothoracic Surgery, Dunedin Hospital, Dunedin, New Zealand; Department of Cardiothoracic Surgery, Dunedin Hospital, Dunedin, New Zealand; Department of Cardiothoracic Surgery, Dunedin Hospital, Dunedin, New Zealand; Department of Infectious Diseases, Dunedin Hospital, Dunedin, New Zealand; Department of Cardiothoracic Surgery, Dunedin Hospital, Dunedin, New Zealand

## Abstract

Pyomyositis is an acute bacterial infection of the skeletal muscle that is commonly associated with localized abscess formation. It is estimated that pyomyositis accounts for up to 4% of all hospital admissions throughout Asia, tropical Africa, Oceania and the Caribbean Islands. However, there has been an increasing emergence of pyomyositis in temperate climates and high-income countries. *Staphylococcus aureus* is the most common organism implicated. Management requires a high index of clinical suspicion, prompt diagnosis and early management to prevent sequalae that can be fatal if left untreated. We describe an interesting case of pyomyositis in an otherwise fit and immunocompetent individual causing mediastinitis; a rare sequalae of the disease. Percutaneous drainage of his left pectoral abscess and a prolonged course of antibiotics provided complete clinical and radiological resolution of the disease despite mediastinal extension. Here we discuss aetiology, associations, pathophysiology and epidemiology of pyomyositis with associated sequalae of the disease.

## INTRODUCTION

Pyomyositis is a purulent infection of skeletal muscle that usually results in abscess formation. The earliest report of pyomyositis dates to Rudolf Virchow in the 1800s. In 1885, Scriba coined the term ‘*tropical pyomyositis’*; a disease that was more common in the tropics [1, 2]. Since then, many cases of pyomyositis have been reported from various geographical regions but the disease remains relatively uncommon in temperate climates [2]. Predisposing risk factors for pyomyositis include immunodeficiency, trauma, intravenous drug use, concurrent infection and malnutrition [3]. Nevertheless, pyomyositis can occur in otherwise healthy individuals without underlying co-morbidities. *Staphylococcus aureus* is the most common bacterial agent [1, 2, 4]. We describe a case of a young man with pyomyositis but a much rarer sequalae of the disease; mediastinitis.

## CASE REPORT

**Figure 1:**
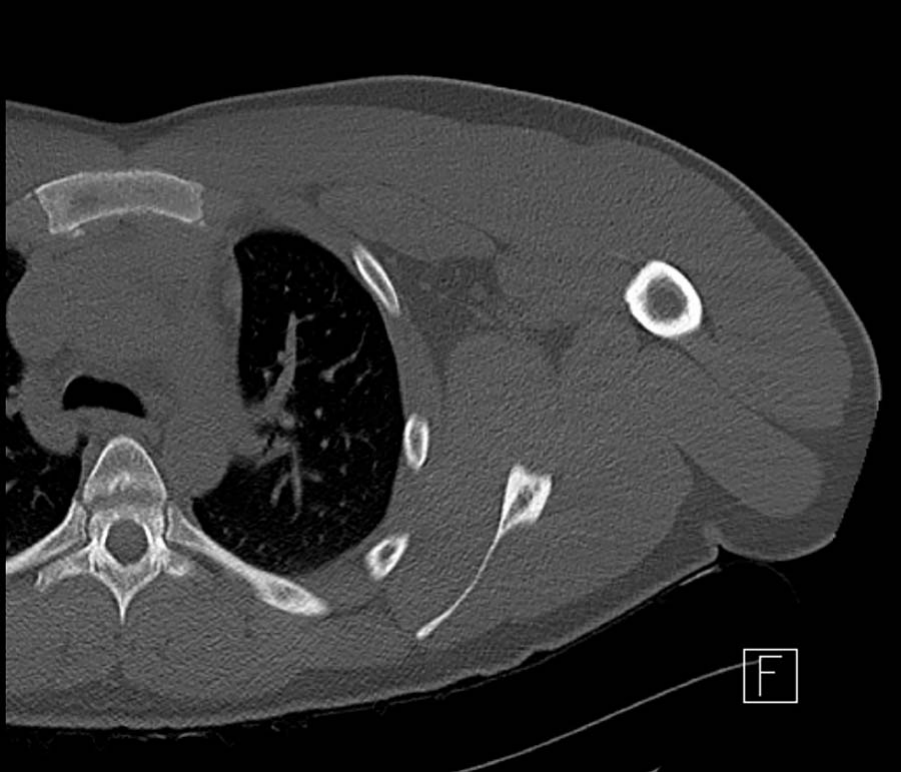
CT scan of left shoulder with no significant soft tissue or mediastinal abnormality.

A 21-year-old man presented to the emergency department complaining of a left shoulder injury after being tackled during a game of rugby 4 days prior. His pain had gradually worsened, and he began feeling non-specifically unwell. He had no other medical history. Examination findings included significant pain and reluctance to move his shoulder with tenderness and swelling overlying his upper left pectoral muscle extending to the sternocostal margin. Computed tomography (CT) of his left shoulder showed no soft tissue or bony abnormality (Fig. 1). He was admitted under the orthopaedic service with concerns of a brachial plexus injury. Overnight he developed a fever, neutrophilia (12 700 cells/μL) and a raised C-reactive protein (CRP) at 126 mg/L.

**Figure 2:**
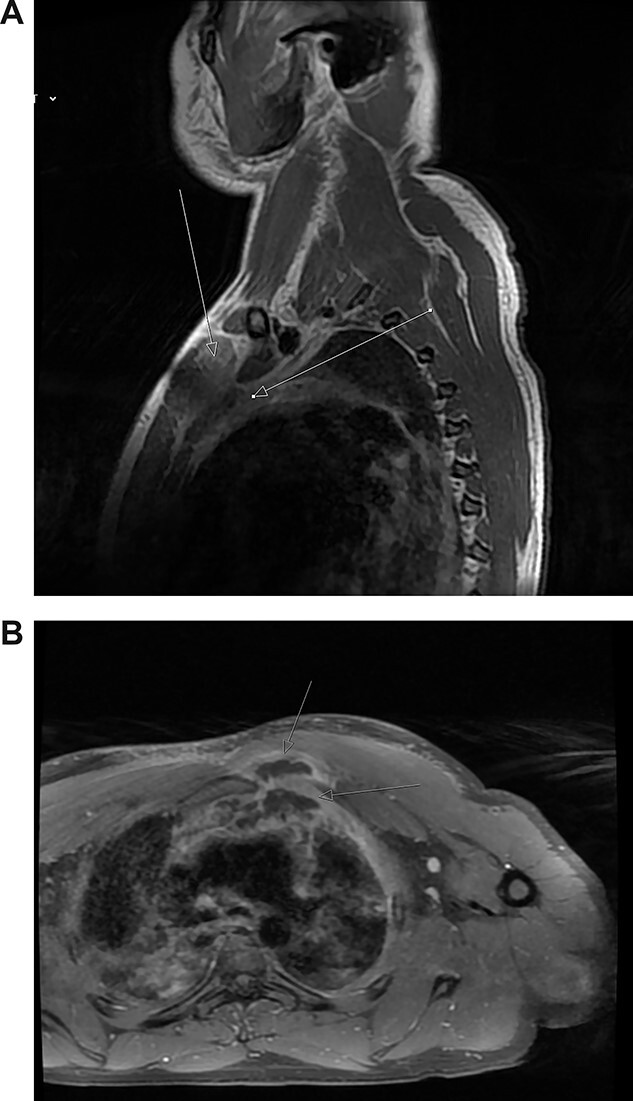
(**A**) Post-contrast MRI scan showing areas enhancement around pectoralis major (myositis) with extension into the mediastinum (mediastinitis) (*white arrows*). (**B**) New rim-enhancing collections within left pectoralis major muscle. The collection appears to extend posteriorly into the anterosuperior mediastinum (*white arrows*)

Magnetic resonance imaging (MRI) performed 1 day after admission showed inflammation and oedema at the superior and medial aspect of the left pectoralis muscle suggesting myositis. This inflammation extended to the left second sternocostal joint, the superior and posterior border of the manubrium abutting the anterior margin of the left brachiocephalic vein to indicate mediastinitis (Fig. 2a). No abnormality was seen in the joints, bone or bone marrow.

The patient was commenced on a regimen of intravenous flucloxacillin and clindamycin. Blood cultures subsequently grew methicillin-sensitive *S. aureus*. Despite antibiotics, his symptoms worsened. His neutrophils and CRP increased to 16 000 cells/μL and 404 mg/L, respectively. Repeat MRI scan on day 5 showed further progression of myositis with new rim-enhancing collections within the left supero-medial pectoralis major muscle extending inferiorly and posteriorly to the first rib sternal articulation. The collection also extended through the left first rib sternal articulation into the anterosuperior mediastinum (Fig. 2b). Furthermore, there was now compression of the left brachiocephalic vein.

**Figure 3:**
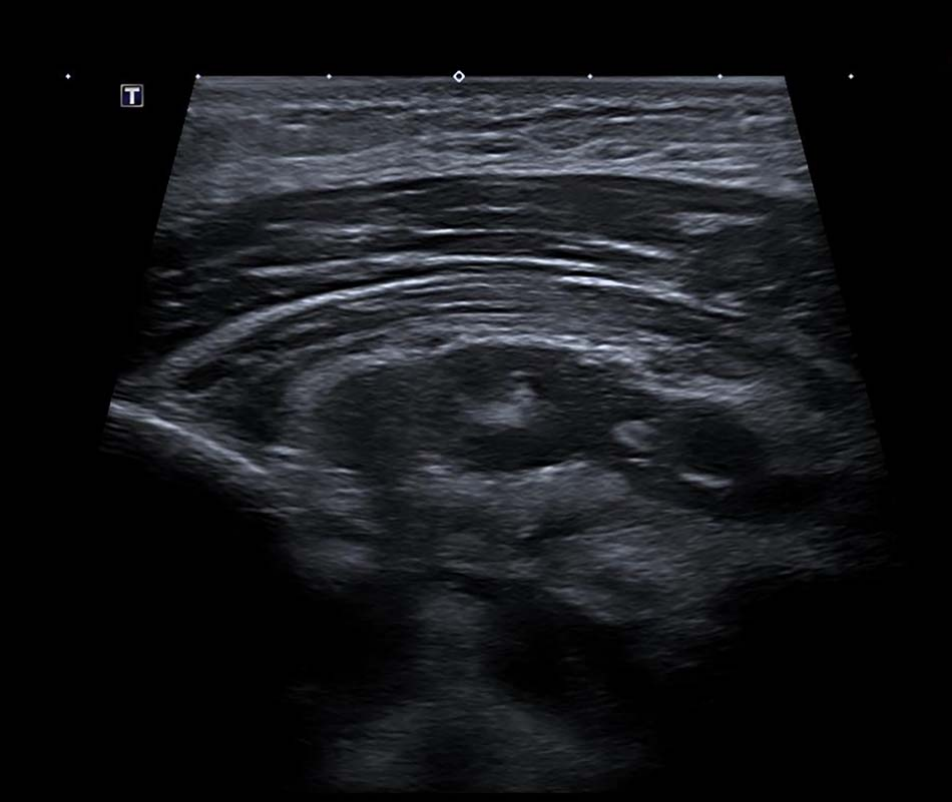
Ultrasound scan demonstrating a retropectoral abscess communicating with the mediastinum.

Under ultrasound guidance, two areas of collection were seen with transmitted cardiac pulsations to suggest communication with the mediastinum (Fig. 3). About 35 ml of thick pus was aspirated. The pectoral abscess grew *S. aureus*. Dual intravenous antibiotics were continued, with significant clinical and biochemical improvement after 1 week. The antibiotic regimen was then switched to intravenous flucloxacillin as a single agent for 4 weeks. He was discharged on day 13 of his admission.

The patient was seen in Infectious Diseases clinic 4 weeks later. His inflammatory markers had completely normalized.

## DISCUSSION

Pyomyositis is a purulent infection of skeletal muscle that usually results in abscess formation. There are various risk factors implicated but perhaps the strongest risk factor is concurrent infection with the Human Immunodeficiency Virus especially in cohorts of patients with Acquired Immunodeficiency Syndrome; a five-time increase in risk [3]. Young males are most susceptible with maximum incidence between 10 and 40 years [2] but all age groups can be affected.

The clinical picture of pyomyositis is divided into three stages. The first stage is the ‘invasive stage’ manifesting as a subacute onset of fever, localized muscle tenderness, swelling, erythema and mild leucocytosis. There may be a preceding history of blunt trauma or vigorous exercise, but it is postulated that a transient period of bacteraemia leads to bacterial proliferation within the iron-rich muscle tissue [2]. The hallmark of the second stage (‘suppurative stage’) is the presence of high swinging fevers and severe systemic symptoms attributing to abscess formation within fascial planes. Aspirating the abscess yields a purulent exudate. In the final stage (‘septicaemic stage’), patients progress to septicaemia, septic shock and may develop metastatic abscesses.

*S. Aureus* is the predominant organism isolated from muscle abscesses in 75–90% of cases [1, 2, 4]. The second most common organism is Group A Streptococcus. Other causative organisms are rare and include streptococcus (groups B, C, G), pneumococcus, haemophilus, aeromonas, serratia, yersinia, pseudomonas, klebsiella and escherichia [2, 5, 6]. There are isolated case studies of pyomyositis caused by salmonella, citrobacter, fusobacterium, mycobacterium, *Neisseria Gonorrhoeae* and nematode larvae [2, 3, 7]. In some cases, no causative organism (sterile pus) is found [1, 6]. The natural history is progressive suppuration with either spontaneous drainage and gradual resolution or eventual bacteraemia and secondary infection [1, 6]. Pyomyositis affects large muscle groups; most commonly the quadriceps femoris, iliopsoas and the gluteus maximus but all skeletal muscle can be affected [1, 2, 6].

Mortality rate from pyomyositis in the late stage is reported to be between 0.89 and 20% [3, 8] but the ‘true’ mortality rate can be somewhat hard to quantify as case series publications only reflect hospital-treated cases. Early diagnosis and treatment are critical to prevent tissue destruction. Non-invasive radiological modalities such as ultrasonography, CT and MRI allow for diagnosis and follow-up. The gold standard remains aspiration of pus from muscle or culture and staining of a muscle biopsy as pyomyositis can masquerade as various clinical entities such as cellulitis, muscle contusion, polymyositis, septic arthritis and osteomyelitis [1, 4, 8].

Aggressive management combining appropriate antibiotics, percutaneous drainage of pus and aggressive surgical debridement may be required. The patient above was managed non-surgically and may highlight how this sequela of mediastinitis may indeed be a different entity to that of descending necrotizing mediastinitis or post-operative mediastinitis which almost exclusively require surgical debridement [9, 10].

There have been only a small number of cases of pyomyositis involving the chest wall. In our patient, we believe that the infected left pectoralis major was due to direct inoculation of the muscle related to trauma. Although chest wall pyomyositis can be associated with osteomyelitis, pneumonia, empyema and septic arthritis [3, 5], a literature search did not bring up a prior report that had association with mediastinitis.
